# Level of completion along continuum of care for maternal, newborn and child health services and factors associated with it among women in India: a population-based cross-sectional study

**DOI:** 10.1186/s12884-021-04198-2

**Published:** 2021-10-27

**Authors:** Ajinkya Kothavale, Trupti Meher

**Affiliations:** International Institute for Population Sciences (IIPS), Mumbai, Deonar 400088 India

**Keywords:** Antenatal care, Continuum of care, India, Institutional delivery, Immunization, Postnatal care

## Abstract

**Background:**

India, being a developing country, presents a disquiet picture of maternal and neonatal mortality and morbidity. The majority of maternal and neonatal mortality could be avoided if the continuum of care (CoC) is provided in a structured pathway from pregnancy to the postpartum period. Therefore, this article attempted to address the following research questions: What is the level of completion along CoC for MNCH services? At which stage of care do women discontinue taking services? and what are the factors affecting the continuation in receiving maternal, newborn and child health (MNCH) services among women in India?

**Methods:**

The study utilized the data from the National Family Health Survey (NFHS-4) conducted during 2015–16 in India. The analysis was limited to 107,016 women aged 15–49 who had given a live birth in the last 5 years preceding the survey and whose children had completed 1 year. Four sequential fixed effect logit regression models were fitted to identify the predictors of completion of CoC.

**Results:**

Nearly 39% of women in India had completed CoC for maternal and child health by receiving all four types of service (antenatal care, institutional delivery, post-natal care and full immunization of their child), with substantial regional variation ranging from 12 to 81%. The highest number of dropouts in CoC were observed at the first stage with a loss of nearly 38%. Further, education, wealth index, and health insurance coverage emerged as significant factors associated with CoC completion.

**Conclusion:**

The major barrier in achieving CoC for maternal and child health is the low utilization of ANC services in the first stage of the continuum and hence should be addressed for increasing CoC completion rate in the country. The gaps across all the levels of CoC indicate a need for increased focus on the CoC approach in India. A strategy should be developed that will connect all the components of MNCH avoiding dropouts and the MNCH provision should be standardized to provide services to every woman and child.

**Supplementary Information:**

The online version contains supplementary material available at 10.1186/s12884-021-04198-2.

## Background

Accelerating progress earlier towards Millennium Development Goals and now towards Sustainable Development Goals to improve the maternal, newborn and child health (MNCH) has achieved high importance on the global agenda [1]. Major investments made by the global community over the past decade have resulted in impressive achievements in reducing maternal, neonatal, and child mortality [2]. The neonatal mortality rate was almost halved from 37 to 18 deaths per 1000 live births between 1990 and 2019 [3], whereas the global maternal mortality rate fell by approximately 38% from 2000 to 2017 [4]. However, many developing countries have failed to achieve that and are still struggling with high mortality burden. Everyday, about 810 maternal deaths happen largely as a result of preventable and treatable causes [3].The majority of deaths occur during pregnancy, delivery and postnatal periods [5, 6]. Similarly, 6700 children under the age of 1 month die every day. In 2019, about 2.4 million newborns died worldwide, and around 1.9 million babies were stillborn [3].

Over the past decades, the World Health Organization (WHO) and other organizations have been advocating for a continuum of care (CoC) to improve maternal, newborn and child health. The CoC is a core organizing principle for health systems, emphasizing the delivery of health care packages throughout time and at different levels of service delivery [2, 7, 8]. An effective CoC addresses the health needs of the woman before, during and after her pregnancy, as well as infant and child care throughout the life cycle, [9]. The advantage of CoC is that each stage builds on the accomplishments of the preceding stage, ensuring a better comprehensive health care experience for women and children [10]. For instance, anFtenatal care (ANC) visits to a healthcare center can help avoid complications throughout pregnancy, resulting in safe and healthy birth [11]. Appropriate skilled care before, during and immediately following childbirth can reduce the risk of death for both the mother and the newborn [7]. However, lack of appropriate care at either of the CoC stages is associated with poor MNCH outcomes [12–14].

The continuum of care emphasizes on two key dimensions that is time and place. The time dimension highlights the importance of linkages among the packages of MNCH service delivery over time at different stages of pregnancy, childbirth, and postpartum periods [7]. It also reflects the need for optimum healthcare when the risk of complications is highest for both mother and newborn. According to a study conducted by [10], more than 50% of all maternal and neonatal mortality occurs during birth and in the first few days of life. Therefore, this study has paid attention to the time dimension for continuity of care at each level of MNCH services.

In general, MNCH services are evaluated separately in terms of antenatal coverage, institutional delivery, postnatal care, full immunization of the child etc. Over the previous few decades, India’s coverage of these services has vastly increased [15]. Several intervention programmes like ‘Janani Suraksha Yojana’ (JSY) and others have increased utilization of maternal and child health services at state and national level. Moreover, a study by James et al. [16] has reported an increase of 25% in the CoC of maternal health care from 2005 to 06 to 2015–16 in India. However, progress in reducing perinatal and neonatal mortality has been modest [17, 18]. According to the report of the National Family Health Survey (NFHS-4), ANC coverage and institutional deliveries were 84% and 79%, respectively. However, coverage of postnatal care and immunization was comparatively less i.e., 65% and 62% respectively [15]. Nevertheless, according to a recent study, only 19% of Indian women have completed the maternal health continuum [19]. These percentages indicate that greater coverage of any individual service does not guarantee that women receive all other required components; this gap is likely to result in continuously worse health outcomes of both mother and the child. Therefore, it is critical that mothers and children receive all necessary health services in a continuum.

Taking all these factors into consideration, this article attempts to address two research questions: (1) What is the level of completion along CoC for MNCH services? At which stage of care do women discontinue taking services? (2) What are the factors affecting the continuation of receiving MNCH services among women in India? Previous research on CoC in India has mostly concentrated on the continuum of maternal healthcare services (antenatal care, institutional care and postnatal care) [16, 19]. However, in order to improve the health condition of children, it is crucial to include interventions regarding child healthcare services in the CoC. Further, the MNCH strategy in India mainly focuses on interventions aimed at several phases of the mother and child’s life cycle, such as antenatal care, delivery care, postnatal care for both mother and their newborn and child vaccination. Therefore, services like 4+ ANC, institutional delivery, postnatal care, and immunization were considered as components of CoC in this study.

## Methods

### Data source

The data was drawn from the fourth round of the National Family Health Survey (NFHS-4), conducted during 2015–16 in India. Being a large scale and nationally representative study, the fundamental objective of NFHS is to dispense state and national level estimates on fertility, reproductive health, maternal and child health, family planning services and nutrition etc. NFHS-4 also provides data on various socio-economic variables and information extending to certain facets of the program implemented in the country. Multistage stratified sampling was adopted by NFHS-4 to provide statistics on various indicators for all 640 districts and 35 states and Union Territories (UTs) as per the 2011 Indian Census classification of districts. Two-stage sampling was carried out in rural areas, whereas urban areas underwent a three-stage sampling. Hence, 601,509 households were interviewed in NFHS-4 [15].

The analysis of this study was limited to women of the reproductive age group (15–49 years) who had a live birth during the last 5 years preceding the surveys and whose child had completed 1 year. Women’s questionnaire was used to collect information on various aspects of maternal and child healthcare such as ANC, delivery care, Post-natal care (PNC), immunization etc. In this study, we limited our analysis to 107,016 women and their children.

### Outcome variables

**Fig. 1:**
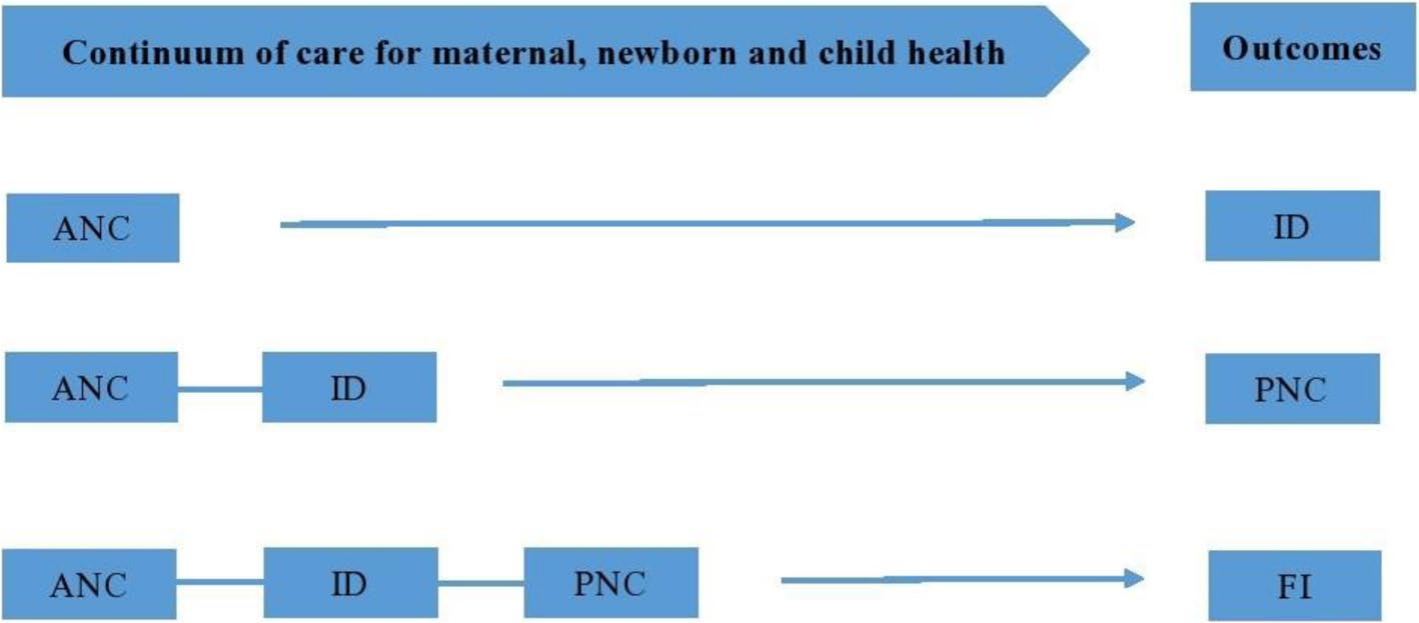
Hypothesized structural relationships for the continuum of care for maternal, newborn and child health

This study used a population-level framework based on integrated service delivery by using four packages such as: antenatal care (ANC), institutional delivery (ID), mother’s post-natal care (PNC) and immunization (FI) to promote health of mothers and children. 4 + ANC was assessed from the question, “How many times you received antenatal care during this pregnancy?” The information regarding ID was obtained from the question: “Where did you give birth to the last child?” and women were considered to have institutional delivery if they had a delivery at hospital, clinic or any other health facility. Furthermore, information on PNC of the mother was obtained from the question: “Did anyone check on your health while you were still in the facility?” Lastly, full immunization was defined based on whether the child aged 12–59 months had received one dose of BCG vaccine, one dose of measles vaccine, three doses of polio and three doses of DPT vaccines [15]. The continuum of care framework for MNCH was adopted from the framework developed by Kerber et al. [7], though, we focused on the utilization of care assuming four stages as shown in Fig. 1.

### Independent variables

The factors included concerning adequate use of MNCH were based on the healthcare utilization framework developed by Andersen [20], which states factors that lead to the use of health services. This framework divides the factors into three groups, which are as follows:

(i) *Presiding factors*: This includes factors like age, which was recoded into five categories as ‘15–24 years’, ‘25–29 years’, ‘30–34 years’, ‘35–39 years’, ‘40–49 years’. Education was categorized as ‘no education’, ‘primary education’, ‘secondary education’ and ‘higher education’. Caste was recoded as ‘Scheduled Castes/Tribes’ (SC/ST), ‘Other Backward Classes’ (OBC), and ‘others’. Religion was recoded as ‘Hindu’, ‘Muslim’ and ‘others’ (Christian/Sikh/Jain/Jewish/Buddhist). Currently married was recoded as ‘yes’, ‘no’ (divorced/widowed/separated). The variable ever used family planning (FP) methods was assessed from the question “Have you ever used anything or tried in any way to delay or avoid getting pregnant?” and was recode as ‘yes’ or ‘no’.
(ii) *Enabling factors***:** This includes factors like wealth index, which was created using household assets and then recoded into five categories such as ‘poorest’, ‘poorer’, ‘middle’, ‘richer’ and ‘richest’. Place of residence was recoded as ‘urban’ and ‘rural’. Health insurance variable was assessed from the question “Are you covered by any health scheme or any health insurance?” and was recoded as ‘yes’ or ‘no’. The mass media exposure variable was derived using three questions: (1)“Do you read a newspaper or magazine almost every day, at least once a week, less than once a week or not at all?” (2) “Do you listen to the radio almost every day, at least once a week, less than once a week or not at all?” (3) “Do you watch television almost every day, at least once a week, less than once a week or not at all?” Responses like 'almost every day' and 'at least once a week' were coded as 1 and rest were as 0. Then the final variable was generated by adding the three variables and was recorded as ‘no’ for 0, ‘low’ for 1, ‘medium’ for 2 and ‘high’ for 3.
(iii) *Risk factors***:** Wanted pregnancy was recoded as ‘wanted then’, ‘wanted later’ and ‘no more’. Ever terminated pregnancy was recoded as ‘yes’ or ‘no’. Pregnancy complications was recoded as ‘yes’ or ‘no’. Ever had a child who died was recoded as ‘yes’ or ‘no’ and sex of the child was recoded as ‘male’ and ‘female’. A description of the study sample segregated by the selected covariates is presented in Table 1**.**

**Table 1:** Descriptive statistics of the study sample of women for the most recent birth, India, NFHS-4, 2015–16

Background characteristics	Weighted % [Frequency]
**Presiding factors**	
**Age (in yrs.)**	
15–24	29.7 [29647]
25–29	40.5 [42251]
30–34	20.4 [23040]
35–39	7.3 [9031]
40–49	2.1 [3047]
**Caste**	
SC/ST	31.2 [40498]
OBC	44.6 [43375]
Other	24.1 [23143]
**Religion**	
Hindu	80.9 [80617]
Muslim	13.5 [13265]
Other	5.6 [13134]
**Mothers’ education**	
No Education	22.4 [25447]
Primary	13.4 [14832]
Secondary	50.4 [53515]
Higher	13.9 [13222]
**Currently married**	
Yes	98.5 [104992]
No	1.5 [2024]
**Ever used FP method**	
No	36.7 [40316]
Yes	63.3 [66700]
**Enabling factors**	
**Place of residence**	
Urban	33.6 [30803]
Rural	66.4 [76213]
**Wealth Index**	
Poorest	17.6 [20545]
Poorer	19.9 [22593]
Middle	20.9 [22645]
Richer	21.6 [21242]
Richest	19.9 [19691]
**Had health insurance**	
No	82.4 [89640]
Yes	17.6 [17376]
**Mass media exposure**	
No	25.3 [29869]
Low	48.3 [50339]
Medium	22.2 [22833]
High	4.2 [3975]
**Risk factors**	
**Wanted pregnancy**	
Then	92.3 [98651]
Later	3.5 [3913]
No more	4.2 [4452]
**Ever terminated pregnancy**	
No	82.6 [88245]
Yes	17.4 [18771]
**Pregnancy complications**	
No	36 [40153]
Yes	64 [66863]
**C section delivery**	
No	78.4 [88434]
Yes	21.6 [18582]
**Ever had a child who died**	
No	91.8 [97588]
Yes	8.2 [9428]
**Sex of child**	
Male	55.3 [58992]
Female	44.7 [48024]

### Statistical analyses

All the analyses were conducted using STATA version 15.0. Descriptive statistics of selected socio-demographic and other characteristics were performed. Four sequential fixed effect logit regression models were fitted to identify the predictors of CoC. To identify the determinants of ANC at the stage of pregnancy, Model I was fitted among all studied women with receiving four or more ANC as the outcome variable. Among those women who received ANC, some went for ID and some did not. So, Model II was fitted among those women who continued from ANC to ID. Further, we fitted Model III among those women who had received ANC, ID and PNC. Finally, Model IV was fitted for those who received ANC, ID, PNC and had also taken their children for FI.

The models were of the form

$${\mathrm{Y}}_{\mathrm{ij}}={\mathrm{O}}_{\mathrm{j}}+{\mathrm{k}}_{\mathrm{j}}\left({\mathrm{X}}_{\mathrm{ikj}}\right)+{\mathrm{e}}_{\mathrm{ij}}$$

Where Y_ij_ denotes the log odds of a woman i in a cluster (community) j continuing at any stage of the maternity care continuum; O_j_ is the intercept for individual-level model (average risk of continuing at any stage in cluster j); X_ikj_ is the covariates (education, age group, wealth index, etc.); k_j_ is the coefficients for the individual level covariates; e_ij_ is the error terms for the individual-level model. We then calculated the intra-cluster correlation (ICC (ρ)) in the dependent variable for stages I to IV. The ρ value indicates the proportion of the total variance at the cluster level. Latent variable method [21] is used to calculate ICC; as shown below:

$$\uprho =\frac{\sigma_1^2}{\sigma_1^2+{\pi}^2/3}$$

Where σ_1_^2^ is the variance between clusters and π^2^/3 is the estimated variance between individuals. We then calculated the proportion of the cluster-level variance that is explained by different blocks of covariates as follows:

$${\sigma}_e^2=\frac{\sigma_i^2-{\sigma}_{ii}^2}{\sigma_i^2}$$

Where σ_e_^2^ is the explained variance, σ_i_^2^ is the variance in the initial or empty model, and σ_ii_^2^ is the second-level variance in the models with various blocks of covariates. Fixed effects logistic regression model is used for controlling unobserved heterogeneity if present in the study sample. Adjusted odds ratio with 95% confidence interval were calculated to assess the strength of the association of MNCH indicators with covariates. All the estimates provided in this study were derived by applying appropriate sampling weights provided by NFHS-4.

## Results

### Utilization of MNCH services

Table 2 depicts that in India, nearly 62% of women had the WHO-recommended four or more ANC visits. About 75% of women were supervised by a skilled birth attendant (SBA) at the time of delivery. Further, 85.5% of women had their deliveries in a healthcare center and out of them, 53.5% were reported that they had their deliveries in public healthcare centers whereas 32% had their delivery in private hospitals. More than three-fourth of the women had received post-natal care. Furthermore, approximately 75% children had received full immunization.

**Table 2:** Percentage of maternal, newborn and child health services received by women and children, India, NFHS-4, 2015–16

Type of services	Prevalence (%)	N
**Antenatal care visit**		
No visit	1.04	1314
One	6.58	7082
Two	14.54	17,525
Three	16.04	20,130
Four or more	61.8	60,965
**Birth attendance**		
Doctor	27.51	24,556
Nurse/ANM	45.86	51,937
Other health personnel	1.57	1620
Traditional health worker	7.95	9517
Relative/friend	17.1	19,386
**Place of delivery**		
Public hospital	53.49	61,424
Private hospital	32.0	27,498
Home/other than health facility	14.0	18,094
**Post-natal care**		
No	23.3	27,056
Yes	76.7	79,960
**Full immunization**		
No	24.94	27,749
Yes	75.06	79,267

### Continuum of care

**Fig. 2:**
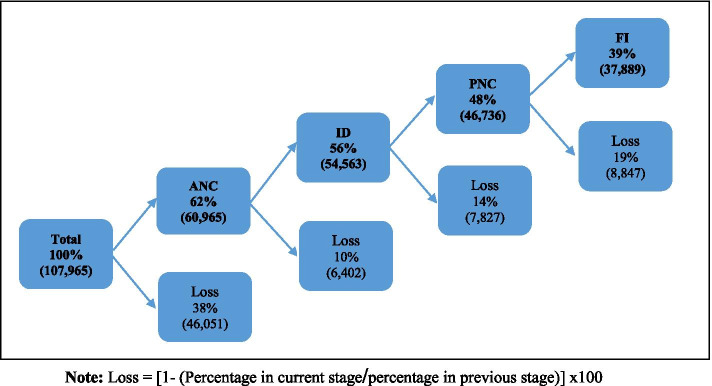
Flowchart of the continuum of care across the maternal, newborn and child healthcare of women for the most recent birth, India, NFHS, 2015–16

Figure 2 presents a flowchart that shows the percentage of CoC of MNCH in India as a whole and the loss at each stage. Among all the sampled women, about 62% had used 4 + ANC services. However, 56% of women retained in the continuum of care at delivery stage, while 10% were lost from the continuum. Further, out of those women who had institutional delivery along with 4 + ANC visits, about 48% had gone for PNC with a loss of 14% at third level. In the end, only 39% had completed CoC, who were defined as the women who received adequate ANC, ID, PNC and also taken their children for full immunization. The last stage of CoC also recorded a loss of 19%. The highest number of dropouts in CoC were observed at the first stage with a loss of nearly 38%. However, the use of services varies substantially by background characteristics (Supplementary Table S2).

Table 3 shows the combination of MNCH services that women and their children had received. As per the results, only 38.8% had received all four types of services, whereas 2.3% had not received any of the services. A total of 7.5% had received only one of the four types of care. Similarly, 17.4% availed only two types of health care services and did not go for the rest. Further, around 34% had received three out of four mentioned services. However, 16.2% of women had received ID and PNC and also full immunization for their children without first having ANC.

**Table 3:** Percentage distribution of different types of maternal, newborn and child health services received by women and children, India, NFHS-4, 2015–16

Adequate antenatal care visit (ANC)	Institutional delivery (ID)	Post-natal care (PNC)	Full immunization (FI)	%
–	–	–	–	**2.3**
–	–	–	+	**3.1**
–	–	+	–	**1.1**
–	–	+	+	**2.1**
–	+	–	–	**2.5**
–	+	–	+	**4.7**
–	+	+	–	**6.2**
–	+	+	+	**16.2**
+	–	–	–	**0.8**
+	–	–	+	**1.7**
+	–	+	–	**0.6**
+	–	+	+	**2.4**
+	+	–	–	**2.1**
+	+	–	+	**6.1**
+	+	+	–	**9.3**
+	+	+	+	**38.8**
**Total**				**100**

+ received the service; − Did not receive the service

Table 4 shows the states/UTs-wise conditional prevalence of each stage of CoC of MNCH services. States like Bihar, Jharkhand, Uttar Pradesh, Nagaland, and Arunachal Pradesh had shown a consistently low utilization of MNCH services, whereas Goa, Kerala, and Tamil Nadu had shown high utilization of services at each level. In this study sample, the percentage of 4 + ANC coverage varied from 25.9% in Bihar to 95.4% in Andaman & Nicobar Islands. The conditional prevalence of institutional delivery was highest in Kerala (100%) followed by Puducherry (99.8%) whereas, the lowest conditional prevalence was observed in Meghalaya (73.2%). Furthermore, Arunachal Pradesh had shown the lowest conditional prevalence for PNC service. However, in the case of full immunization, Chandigarh had reported the highest conditional prevalence i.e.,  95.9%. From the table it is quite clear that the level of CoC was highest among the southern states of India.

**Table 4:** State/UT wise conditional prevalence of maternal, newborn and child health care services received by women for the most recent birth, India, NFHS-4, 2015–16

State and Union territories	Region	ANC	ANC and ID	ID and PNC	PNC and FI	ANC and ID and PNC and FI
Andaman & Nicobar Islands	Southern	95.41	97.20	77.60	72.25	52.80
Andhra Pradesh	Southern	75.75	92.33	88.69	78.80	50.50
Arunachal Pradesh	North-eastern	43.20	83.58	64.92	64.77	18.55
Assam	North-eastern	54.94	88.87	82.39	74.87	33.32
Bihar	Eastern	25.89	84.37	71.59	75.88	12.70
Chandigarh	Northern	66.25	94.80	97.24	95.97	58.33
Chhattisgarh	Central	62.20	74.72	82.31	88.77	36.97
Dadra & Nagar Haveli	Western	84.21	93.18	86.79	71.66	52.63
Daman & Diu	Western	76.29	93.59	75.44	72.72	39.63
Goa	Western	91.47	99.19	94.65	92.78	81.30
Gujarat	Western	82.13	93.73	75.76	69.61	43.69
Haryana	Northern	54.04	92.28	84.38	78.37	35.99
Himachal Pradesh	Northern	74.79	84.82	91.74	80.35	47.72
Jammu And Kashmir	Northern	82.61	88.55	88.78	83.02	55.83
Jharkhand	Eastern	40.03	79.46	70.61	81.07	21.16
Karnataka	Southern	80.74	95.40	74.39	74.96	44.08
Kerala	Southern	90.61	100.00	93.23	89.10	76.14
Lakshadweep	Southern	83.40	98.59	94.77	94.81	73.08
Madhya Pradesh	Central	45.95	91.39	73.40	72.76	24.67
Maharashtra	Western	77.78	93.45	87.26	73.01	48.00
Manipur	North-eastern	77.93	86.08	94.32	80.70	53.46
Meghalaya	North-eastern	60.17	73.22	90.54	77.58	33.31
Mizoram	North-eastern	70.31	95.11	85.32	63.92	38.66
Nagaland	North-eastern	32.70	76.53	72.44	56.35	12.47
Delhi	Northern	75.34	93.54	73.66	87.62	45.19
Odisha	Eastern	65.66	89.98	87.66	85.09	46.04
Puducherry	Southern	89.33	99.83	94.75	91.40	76.94
Punjab	Northern	69.98	94.85	94.52	94.09	60.03
Rajasthan	Northern	45.01	92.89	77.80	74.06	26.60
Sikkim	North-eastern	79.30	95.03	82.34	90.15	56.36
Tamil Nadu	Southern	87.22	99.37	90.90	80.89	64.69
Tripura	North-eastern	72.04	91.29	80.16	67.40	38.83
Uttar Pradesh	Central	34.53	86.63	80.40	66.76	19.12
Uttarakhand	Central	39.76	86.70	80.40	79.72	24.55
West Bengal	Eastern	84.32	82.25	78.19	90.96	51.70
Telangana	Southern	77.10	93.72	91.32	76.23	50.57
**India**		**61.8**	**91.01**	**82.07**	**77.59**	**38.83**

Conditional prevalence represents the prevalence at each stage of CoC given that the respondent already achieved previous stage

### Results from regression analysis

Table 5 depicts the inhibiting factors for women to receive the MNCH services. Model I analysed the correlates of women using 4 + ANC services. Results revealed that all factors except marital status were significantly associated with the use of 4 + ANC. Women living in rural areas were 31% (AOR = 0.69; CI: 0.66–0.74) less likely to use 4 + ANC services as compared to urban women. Further, factors like higher educational level, higher wealth index, use of family planning methods, health insurance, mass media exposure etc. had shown higher odds of 4 + ANC visits. In addition, women with pregnancy complications (AOR = 1.88; CI: 1.52–1.95) and history of terminated pregnancy (AOR = 1.14; CI: 1.10–1.19) were more likely to receive 4 + ANC. Moreover, women having health insurance coverage had higher odds (AOR = 1.42; CI: 1.35–1.49) of receiving 4 + ANC than their counterparts. In Model I, ρ value was 0.38, which means that between-cluster variation accounts for just 38% of the total variation in receiving full antenatal care and the remaining 62% of the variation was caused by individual characteristics.

**Table 5:** Predictors of continuum of care across the maternal, newborn and child health care for women and children, India, NFHS-4, 2015–16

Background characteristics	Model I (***N*** = 107,016)		Model II (***N*** = 60,965)		Model III (***N*** = 54,563)		Model IV (***N*** = 46,736)	
	ANC		ANC & ID		ANC & ID & PNC		ANC & ID & PNC & FI	
	AOR	95% CI	AOR	95% CI	AOR	95% CI	AOR	95% CI
**Age groups (15–24 yrs.)®**								
25–29 yrs.	1	0.96–1.04	0.8^c^	0.71–0.91	1.05	0.97–1.14	1.08	1–1.16
30–34 yrs.	1	0.95–1.05	0.75^c^	0.64–0.87	1.02	0.92–1.12	1.07	0.99–1.17
35–39 yrs.	0.99	0.93–1.06	1.02	0.81–1.27	1.05	0.92–1.21	1.08	0.96–1.21
40–49 yrs.	0.98	0.88–1.09	0.69	0.5–0.95	1.1	0.87–1.39	1.05	0.87–1.28
**Education (No)®**								
Primary	1.23^c^	1.16–1.3	0.98	0.83–1.16	0.93	0.82–1.05	1.14^c^	1.02–1.27
Secondary	1.62^c^	1.54–1.7	1.43^c^	1.23–1.65	1.01	0.91–1.12	1.46^c^	1.33–1.6
Higher	2.11^c^	1.96–2.27	2.18^c^	1.69–2.82	1.19^a^	1.03–1.37	1.54^c^	1.36–1.74
**Caste (SC/ST)®**								
OBC	0.94^a^	0.9–0.98	1.2	1.05–1.37	0.92	0.84–1	1.02	0.94–1.09
Other	1.09^c^	1.03–1.15	1.08	0.93–1.26	0.92	0.84–1.02	1	0.92–1.09
**Religion (Hindu)®**								
Muslim	1.02^c^	0.96–1.09	0.73^c^	0.61–0.86	1.1	0.97–1.23	0.72^c^	0.66–0.8
Other	0.96^c^	0.9–1.03	0.67^c^	0.57–0.79	1.22^c^	1.08–1.39	0.9^c^	0.82–1
**Currently married (Yes)®**								
No	1.1	0.98–1.24	1.23	0.84–1.81	1.01	0.8–1.27	0.79^a^	0.65–0.96
**Ever used FP method (No)®**								
Yes	1.2^c^	1.16–1.25	0.97	0.87–1.09	1.42^c^	1.33–1.53	1.47^c^	1.38–1.57
**Wealth Index (Poorest)®**								
Poorer	1.37^c^	1.3–1.45	1.35^c^	1.14–1.59	1	0.88–1.13	0.94	0.84–1.06
Middle	1.7^c^	1.6–1.81	1.7^c^	1.42–2.04	1.11	0.97–1.27	1.02	0.9–1.15
Richer	2.06^c^	1.92–2.21	1.84^c^	1.5–2.25	1.16^a^	1.01–1.34	1.06	0.93–1.2
Richest	2.54^c^	2.35–2.76	2.47^c^	1.94–3.15	1.2^a^	1.02–1.4	1.34^c^	1.16–1.55
**Place of residence (Urban)®**								
Rural	0.69^c^	0.66–0.74	0.87^c^	0.75–1	1.19^c^	1.08–1.31	1.15^c^	1.06–1.24
**Had health insurance (No)®**								
Yes	1.42^c^	1.35–1.49	0.91	0.8–1.05	1.09^c^	1–1.19	1.33^c^	1.23–1.44
**Mass media exposure (No)®**								
Low	1.48^c^	1.41–1.55	1.05	0.91–1.21	1.26^c^	1.14–1.39	1.2^c^	1.1–1.32
Medium	1.76^c^	1.65–1.87	1.14	0.95–1.37	1.53^c^	1.35–1.73	1.14^c^	1.02–1.27
High	1.93^c^	1.74–2.14	1.32	0.94–1.85	1.45^c^	1.2–1.74	1.09	0.93–1.27
**Wanted pregnancy (Then)®**								
Later	0.85^c^	0.78–0.92	0.87	0.66–1.15	1.05	0.88–1.25	0.9	0.77–1.05
No more	0.62^c^	0.57–0.68	0.6^c^	0.47–0.76	0.81^a^	0.67–0.98	0.69^c^	0.59–0.82
**Ever terminated pregnancy (No)®**								
Yes	1.14^c^	1.1–1.19	0.91	0.8–1.05	1.25^c^	1.14–1.37	0.97	0.9–1.04
**Pregnancy complications (No)®**								
Yes	1.88^c^	1.82–1.95	1.09	0.98–1.23	1.68^c^	1.56–1.8	1.26^c^	1.18–1.34
**Ever had a child who died (No)®**								
Yes	0.89^c^	0.84–0.94	0.76^c^	0.64–0.91	1.19^a^	1.03–1.36	0.93	0.83–1.05
**Type of birth attendant (Doctor)®**			1.17^a^	1.04–1.31				
Nurse/ANM			0.25^c^	0.2–0.32	1.34^c^	1.25–1.44	1.17^c^	1.1–1.25
Other					2.28^c^	1.75–2.97	1.53^c^	1.24–1.88
**C section delivery (No)®**								
Yes					3.11^c^	2.84–3.4	1.03	0.96–1.1
**Sex of child (Male)®**								
Female					0.99	0.92–1.05	1.05^c^	0.99–1.11
**Child age (12–23 month)®**						–		–
24–47 month					0.93^c^	0.87–1	1.17^c^	1.1–1.24
48–59 month					0.91^c^	0.82–1	1.11^b^	1.02–1.21
**ρ (Rho)**	**0.38**	**0.37–0.39**	**0.38**	**0.34–0.42**	**0.47**	**0.45–0.49**	**0.31**	**0.29–0.33**

*AOR* Adjusted Odds Ratio, *CI* Confidence Interval, *ANC* Antenatal care, *ID* Institutional delivery, *PNC* Post natal care, *FI* Full immunization

®: reference category; ^a^, ^b^, ^c^ refers to < 0.1, < 0.05, < 0.01 level of significance

Model II analysed the correlates of using four or more ANCs as well as institutional delivery services. The results showed a positive association of education and wealth index with 4 + ANC and institutional delivery. The odds of receiving 4 + ANC and institutional delivery increased with the increase in educational attainment and wealth index. However, women from rural areas were significantly less (AOR = 0.87; CI: 0.75–1.00) likely to have 4 + ANC and institutional delivery. ρ value for this model was also 0.38. Model III explores the association of correlates on the continuity of care from delivery to the post-delivery period among women who had already received 4 + ANC and ID. All the covariates found to be significant in Model I also remained significant in the third model except for caste. Besides, increased odds were observed for women who had a c-section delivery (AOR = 3.11; CI: 2.84–3.40).

According to Model IV, factors like higher education, higher wealth quintiles, use of family planning methods, rural residence, mass media exposure, history of pregnancy complications were significantly associated with CoC completion. ρ value for this model was 0.31, which indicated that 69% of the variations were due to individual characteristics.

## Discussion

The continuum of care has become an important approach for reducing maternal, newborn and child mortality. Women’s reproductive age, pregnancy, infancy and childhood are the crucial stages impinging MNCH. As CoC promotes integrated MNCH services, an improvement in MNCH services has become the explicit focus of each country, including India, in identifying and understanding the gaps in seeking healthcare along the pathway of CoC [11]. Therefore, the present study has examined the level of CoC in India considering the utilization of four major aspects of MNCH services such as 4 + ANC, institutional delivery, postnatal care and immunization as well as their association with various individuals and household related factors.

This study revealed that most of the women and their newborns did not receive MNCH services continuously. As per the results, only 38.8% of women in India have completed CoC for maternal as well as child health at all four levels, which also means the level of CoC for the country is 38.8%. This low completion rate of CoC in the country suggests a higher risk of infant and neo-natal mortality, as many women and their children could miss proven interventions at various contact points of the continuum. However, the percentage is higher than in the studies conducted in Ethiopia, Tanzania, Cambodia, Pakistan and nine sub-Saharan African and South Asian countries [22–26], whereas lower than in a study conducted in Nepal [27]. The possible reasons behind such a difference in the level of CoC could be socio-cultural differences, time variation and variation in sources of data. Further, the southern states of India have shown a comparatively higher level of CoC. More efficient health care system, better awareness of healthcare services, and favourable attitudes towards the necessity of mother and child health care in the Southern region of India could be the contributing factors for this result [28, 29].

Our research also found that one of the most significant barriers to obtain CoC for MNCH services in the nation is the poor uptake of 4 + ANC visits. The highest number of dropouts in CoC were also observed at the first stage with a loss of nearly 38%. ANC is an essential area for initial contact with regard to a continuation through CoC [30]. However, ANC utilization is inadequate and inequitable in India. Receiving antenatal care is considered as a significant predictor of subsequent use of skilled assistance during delivery [31]. This makes women better informed about pregnancy and recognizes the importance of skilled birth attendance, institutional delivery, post-natal care and thus improve the continuity of care [32, 33]. Therefore, based on the effects of adequate ANC visits on subsequent maternal health services, it seems right to consider ANC as a powerful tool that connects the other indicators of maternal and child health services. Further, factors like higher maternal education, higher wealth index and exposure to mass media were found to be significantly associated with greater odds of 4 + ANC utilization. This result is consistent with the findings of previous research [34, 35].

In accordance with previous studies [16, 19, 24], our results also indicated a positive association between the educational level of women and CoC completion. According to the analysis, the likelihood of receiving all four MNCH services in continuum was significantly higher among women with high educational attainment. Several literature has also documented the positive impact of education on the utilization of maternal healthcare services [36–38]. This could be because education can help women grasp the notion of safe motherhood and enhances the chances of obtaining high-quality and comprehensive maternal health care. Further, the findings of this study showed that women belonging to the richest wealth quintile were more likely to complete CoC. This result is in line with the findings of previous research [23, 39]. The significant rich–poor disparity in CoC completion might be explained by the lack of affordability. It also raises questions on the potential of the programmes like JSY to increase demand for MNCH services among impoverished women. Our study documented a significant association between health insurance and CoC completion. The significance of health insurance membership in increasing access to maternal and child health care has also been highlighted by Bosomprah et al. [40].

As the study is based on the data from a large-scale nationally representative survey in India, one of the major strengths is the wider relevance of its results. The study also suffers from several limitations that should be considered while interpreting the results. Firstly, owing to the cross-sectional nature of this study, no causal inference can be established based on these findings. A cohort study would be invaluable in validating our results and understanding the multiple possible effects examined in the present study. Secondly, the self-reported nature of the data can be subjected to reporting or recall biases. Thirdly, it does not include information about the quality of services received. Fourthly, it lacked information on the accessibility and availability of healthcare services and healthcare providers, which led to the discontinuation of CoC. Lastly, the measurement of PNC did not cover the care that women and newborns received throughout the postpartum period, although care in the postpartum period is important to ensure that women and newborns survive and live with a healthy status.

## Conclusions

In conclusion, the current study demonstrated a low rate of continuity in MNCH services in India. The study also found a strong association of factors like education, wealth index and health insurance coverage with the completion of continuum of care for MNCH services. In addition, the major barrier in achieving CoC for maternal and child health is the low utilization of ANC services in the first stage of the continuum and hence should be addressed to increase the CoC completion rate. However, despite many efforts by the government of India to ensure full ANC, there are still some areas where the level of adequate ANC is low. The gaps across all the levels of CoC indicate a need for increased focus on the CoC approach in India. A strategy should be developed that will connect all the components of MNCH avoiding dropouts and the MNCH provision should be standardized to provide services to every woman and child.

## Supplementary Information

**Additional file 1 **:**Table S1.** Selection of sample size from the National Family Health Survey, India, 2015–16. **Table S2.** Percentage of women who received ANC, ANC & ID, ANC & ID & PNC and ANC & ID & PNC & FI services received by background characteristics, India, NFHS-4, 2015–16.

## Data Availability

The data used in this research is publicly available on DHS measures website. Any individual can register and easily obtained data in electronic version from the following website The DHS Program - India: Standard DHS, 2015-16 Dataset.

## Abbreviations

CoC: Continuum of care
MNCH: Maternal, Newborn and Child Health
NFHS: National Family Health Survey
ANC: Antenatal care
SBA: Skilled birth attendance
ID: Institutional delivery
PNC: Post-natal care
FI: Full immunization
UT: Union territories
