# Supplementary material for: Level of completion along continuum of care for maternal, newborn and child health services and factors associated with it among women in India: a population-based cross-sectional study
Source: BMC Pregnancy Childbirth. 2021 Oct 27;21:731. doi: 10.1186/s12884-021-04198-2 (PMC8554854; doi:10.1186/s12884-021-04198-2)
Supplement: Supplementary file 1 — Additional file 1 :Table S1. Selection of sample size from the National Family Health Survey, India, 2015–16. Table S2. Percentage of women who received ANC, ANC & ID, ANC & ID & PNC and ANC & ID & PNC & FI services received by background characteristics, India, NFHS-4, 2015–16. [file 12884_2021_4198_MOESM1_ESM.docx]

**Supplementary file**

**Table S1:** Selection of sample size from the National Family Health Survey, India, 2015-16

| Sr. no | Exclusion criteria | Sample |
| --- | --- | --- |
| 1 | Total interviewed women | 699,686 |
| 2 | Women who have not delivered a live birth in the 5 years preceding the survey | 440,070 |
| 3 | Women who delivered a live birth twice in the last 5 years (we selected most recent delivery only) | 68,729 |
| 4 | Women whose child was survived but not alive at the time of interview | 5,797 |
| 5 | Women who currently does not live with her child | 891 |
| 6 | Women whose child is less than one year | 46,019 |
| 7 | Missing cases for any of the MNCH indicator | 806 |
| 8 | Missing cases for any of the selected covariates | 30,358 |
| Total | **Final sample size** | **107,016** |

**Table S2:** Percentage of women who received ANC, ANC & ID, ANC & ID & PNC and ANC & ID & PNC & FI services received by background characteristics, India, NFHS-4, 2015-16

| **Background characteristics** | **ANC** | **ANC & ID** | **ANC & ID & PNC** | **ANC & ID & PNC & FI** |
| --- | --- | --- | --- | --- |
|  | **% [N]** | **% [N]** | **% [N]** | **% [N]** |
| **Age (in yrs.)** |  |  |  |  |
| 15-24 | 62.54 [16678] | 56.92 [15078] | 48.17 [12715] | 38.54 [10141] |
| 25-29 | 63 [24495] | 57.47 [22003] | 49.41 [18884] | 40.1 [15429] |
| 30-34 | 61.66 [13366] | 56.32 [11927] | 48.17 [10312] | 39.05 [8420] |
| 35-39 | 56.47 [4940] | 50.95 [4348] | 43.84 [3773] | 35.39 [3065] |
| 40-49 | 48.24 [1486] | 40.97 [1207] | 35.45 [1052] | 28.53 [834] |
| **Mothers’ education** |  |  |  |  |
| No education | 41.59 [9923] | 33.1 [7744] | 27.26 [6398] | 20.4 [4812] |
| Primary | 55.75 [7391] | 46.28 [6069] | 37.88 [5054] | 29.22 [3953] |
| Secondary | 68.11 [33851] | 63.77 [31205] | 54.77 [26773] | 44.8 [21960] |
| Higher | 77.35 [9800] | 75.92 [9545] | 67.32 [8511] | 56.23 [7164] |
| **Caste** |  |  |  |  |
| SC/ST | 59.19 [22049] | 51.95 [18873] | 44.3 [16158] | 36.25 [12993] |
| OBC | 59.66 [23986] | 55.2 [21953] | 47.52 [18708] | 38.16 [15164] |
| Other | 69.14 [14930] | 63.73 [13737] | 54.05 [11870] | 43.43 [9732] |
| **Religion** |  |  |  |  |
| Hindu | 61.49 [45608] | 56.52 [41300] | 48.23 [35213] | 39.25 [28800] |
| Muslim | 59.54 [7389] | 50.99 [6348] | 42.67 [5403] | 31.91 [4139] |
| Other | 71.65 [7968] | 64.91 [6915] | 59.09 [6120] | 49.47 [4950] |
| **Currently married** |  |  |  |  |
| Yes | 61.76 [59796] | 56.23 [53536] | 48.1 [45865] | 38.87 [37244] |
| No | 64.39 [1169] | 57.21 [1027] | 47.19 [871] | 36.57 [645] |
| **Ever used FP method** |  |  |  |  |
| No | 56.12 [20920] | 51.35 [18575] | 42.53 [15342] | 32.48 [11718] |
| Yes | 65.1 [40045] | 59.08 [35988] | 51.31 [31394] | 42.52 [26171] |
| **Wealth index** |  |  |  |  |
| Poorest | 38.42 [7434] | 28.56 [5496] | 23.37 [4509] | 18.27 [3504] |
| Poorer | 54.26 [11316] | 46.11 [9498] | 38.06 [7907] | 30.09 [6228] |
| Middle | 64.66 [13498] | 59.52 [12203] | 50.71 [10430] | 40.26 [8386] |
| Richer | 71.22 [14197] | 67.7 [13320] | 58.85 [11519] | 47.48 [9314] |
| Richest | 76.75 [14520] | 74.94 [14046] | 65.5 [12371] | 54.84 [10457] |
| **Place of residence** |  |  |  |  |
| Urban | 73 [21084] | 69.44 [19894] | 59.67 [17139] | 48.02 [13898] |
| Rural | 56.12 [39881] | 49.55 [34669] | 42.21 [29597] | 34.18 [23991] |
| **Had health insurance** |  |  |  |  |
| No | 59.25 [49497] | 53.94 [44388] | 45.8 [37897] | 36.56 [30402] |
| Yes | 73.72 [11468] | 67 [10175] | 58.79 [8839] | 49.45 [7487] |
| **Mass media exposure** |  |  |  |  |
| No | 39.25 [11162] | 31.07 [8744] | 25.16 [7116] | 18.93 [5408] |
| Low | 66.58 [30592] | 60.7 [27523] | 51.67 [23456] | 41.98 [19152] |
| Medium | 74.58 [16270] | 72.06 [15476] | 63.41 [13674] | 52.24 [11290] |
| High | 74.75 [2941] | 72.68 [2820] | 63.74 [2490] | 51.44 [2039] |
| **Wanted pregnancy** |  |  |  |  |
| Then | 62.64 [56976] | 57.18 [51158] | 48.89 [43848] | 39.61 [35644] |
| Later | 59.15 [2133] | 52.85 [1890] | 45.18 [1631] | 36.83 [1312] |
| No more | 45.69 [1856] | 38.46 [1515] | 32.92 [1257] | 23.43 [933] |
| **Ever terminated pregnancy** |  |  |  |  |
| No | 61.87 [50033] | 56.31 [44774] | 47.93 [38098] | 38.67 [30880] |
| Yes | 61.44 [10932] | 55.93 [9789] | 48.83 [8638] | 39.6 [7009] |
| **Pregnancy complications** |  |  |  |  |
| No | 49.73 [18483] | 44.64 [16125] | 36.86 [13165] | 28.98 [10268] |
| Yes | 68.58 [42482] | 62.76 [38438] | 54.4 [33571] | 44.37 [27621] |
| **C-section delivery** |  |  |  |  |
| No | 57.37 [47127] | 50.28 [40725] | 41.66 [33907] | 33.58 [27377] |
| Yes | 77.86 [13838] | 77.86 [13838] | 71.39 [12829] | 57.88 [10512] |
| **Ever had a child who died** |  |  |  |  |
| No | 63.16 [56797] | 57.85 [51211] | 49.46 [43847] | 40 [35627] |
| Yes | 46.49 [4168] | 38.2 [3352] | 32.68 [2889] | 25.73 [2262] |
| **Age of child** |  |  |  |  |
| Less than 2 yrs. | 60.26 [20420] | 55.15 [18400] | 47.2 [15776] | 37.45 [12534] |
| 2-3 yrs. | 62.27 [29464] | 56.77 [26433] | 48.51 [22639] | 39.61 [18568] |
| 4 yrs. | 63.37 [10810] | 56.91 [9484] | 48.67 [8121] | 39.5 [6638] |
| **Sex of child** |  |  |  |  |
| Male | 61.41 [33393] | 56.13 [29985] | 48.03 [25700] | 38.64 [20782] |
| Female | 62.28 [27572] | 56.39 [24578] | 48.16 [21036] | 39.08 [17107] |
| **Total** | **61.8 [60,965]** | **56.2 [54563]** | **48.1 [46736]** | **38.8 [37889]** |
